# A cultural-ecosocial systems view for psychiatry

**DOI:** 10.3389/fpsyt.2023.1031390

**Published:** 2023-04-13

**Authors:** Ana Gómez-Carrillo, Laurence J. Kirmayer

**Affiliations:** ^1^Division of Social and Transcultural Psychiatry, McGill University, Montreal, QC, Canada; ^2^Culture and Mental Health Research Unit, Lady Davis Institute, Jewish General Hospital, Montreal, QC, Canada

**Keywords:** multilevel explanation, embodiment, enactment, ecosocial, looping effects, cultural psychiatry, clinical case formulation, systems theory

## Abstract

While contemporary psychiatry seeks the mechanisms of mental disorders in neurobiology, mental health problems clearly depend on developmental processes of learning and adaptation through ongoing interactions with the social environment. Symptoms or disorders emerge in specific social contexts and involve predicaments that cannot be fully characterized in terms of brain function but require a larger social-ecological view. Causal processes that result in mental health problems can begin anywhere within the extended system of body-person-environment. In particular, individuals’ narrative self-construal, culturally mediated interpretations of symptoms and coping strategies as well as the responses of others in the social world contribute to the mechanisms of mental disorders, illness experience, and recovery. In this paper, we outline the conceptual basis and practical implications of a hierarchical ecosocial systems view for an integrative approach to psychiatric theory and practice. The cultural-ecosocial systems view we propose understands mind, brain and person as situated in the social world and as constituted by cultural and self-reflexive processes. This view can be incorporated into a pragmatic approach to clinical assessment and case formulation that characterizes mechanisms of pathology and identifies targets for intervention.

## Introduction

Current psychiatric research assumes the mechanisms of mental disorders can be understood in terms of neurobiology, especially brain circuitry. However, mental health problems clearly depend on developmental processes of learning and adaptation through ongoing interactions with the environment. Human environmental niches are socially and culturally constructed. Symptoms or disorders emerge in specific social contexts and predicaments that cannot be fully characterized in terms of brain function but require a larger ecological systems view. Causal processes can begin anywhere in this larger ecosocial system. In particular, individuals’ narrative self-construals, culturally mediated interpretations of symptoms and coping strategies, as well as the responses of others in the social world, can play a crucial role in the mechanisms of mental disorders, illness experience, treatment response, and recovery. In this paper, we outline the conceptual basis and practical implications of this hierarchical systems view for psychiatric theory and practice. We argue for the importance of adopting a *cultural*-*ecosocial systems view* that understands the brain as situated in the social world and as part of larger, self-reflexive systems that are embodied and enacted through language and other cultural practices (1). This view builds on work in systems biology, social epidemiology, developmental psychology, anthropology and 4E cognitive science to provide a process-based view of the dynamic interactions of subjective experience and social context.

We use the term ‘ecological’ here in a way that is related directly to ecology [as the study of biological organisms in relationship to their physical environments (2); see (3, 4)] but with the recognition that for humans, the environments in which we are embedded are fundamentally social and cultural. What flows through these organism-environment systems is not just energy or material (as is the case in typical ecological analysis) but also information, which is essentially relational (5). The material and symbolic (informational) dimensions of our environment are closely related. We inhabit socially constructed niches that enable communication and cooperation (6). We employ cultural knowledge and practices to navigate these niches, which are both local and extended through time and space. In the process, we both actively reconfigure these niches (7) and are reshaped by them at neurobiological, cognitive and social levels (8).

The cultural-ecosocial view encourages us to consider how cognition and experience depend on the dynamics of the system comprising organism and environment. By emphasizing systemic processes, this view takes a step away from narrow concepts of mechanism that assume the total decomposability of a system into its parts (reductionism), with the recognition that the ways that the parts or constituents of systems are spatiotemporally arranged and connected give rise to new dynamics. System dynamics arise from connectivity, organization and interactions not simply from the properties of the components. Interactions between components may change the properties and function of each of the components as well as the dynamics of local and larger networks.

In the sections that follow, we first provide a brief genealogy of systems thinking in psychiatry and outline the specific contributions of the existing frameworks that we draw from. We then discuss the nature of hierarchical organization in biology before turning to a discussion of multilevel explanation in psychiatry. The next section argues that 4E cognitive science can provide a path to multilevel integration through a cultural-ecosocial systems view. We then illustrate with a case vignette how this approach can be applied to integrative clinical case formulation. The cultural-ecosocial systems approach includes patient’s experience, self-understanding and agency, as well as social structural processes, in explanations of symptoms, disorders and distress. Finally, we discuss the implications of our approach for psychiatric practice. We are calling for a change in psychiatric theory, research and practice that resists the reification and over-simplification of mental health problems in terms of discrete diagnostic entities by focusing on system dynamics that include individuals’ experience and meaning-making as well as the social-cultural contexts in which the person is embedded and from which psychiatric disorders emerge.

## Systems thinking in psychiatry

The concept of *system* is associated with Enlightenment views of knowledge and has been a central trope in modernity associated with ideas of order and control (9). However, a more abstract notion of system has served as of way to identify important analogies and formal correspondances among diverse phenomena. In this usage, a system is a structured ensemble of parts or processes (components, actors or agents) that interact in ways that allow the structure to persist over time and exhibit distinctive behavior or dynamics. The recognition that very different kinds of ensembles may display similar dynamics reflecting their organization led to the development of general systems theory (10-12) and cybernetics (13). The application of systems theory received new impetus with the development of computational approaches to modelling dynamics. Mathematical analyses and computational modelling revealed complex dynamics emerging from even simple systems spawning the development of subfields of nonlinear systems theory, and popular re-branding under the banners of “chaos” and “complexity theory” (14–19). The focus on dynamics supports an ontology in which systems are characterized not in terms of their constituent parts and structures but rather in terms of interactional processes (20, 21).

The concepts introduced in general systems theory and cybernetics were applied widely to modelling behavioral, biological, ecological and social-economic systems [for overviews see (10, 22, 23)]. Efforts to understand biological processes at genomic and cellular levels led to the development of systems biology (24). In this view, the function of components of biological systems like genes, organelles, cells, tissues and organs can only be properly understood by considering their relation to the dynamics of the larger system as a whole. Understanding these dynamics holds great promise for improving clinical approaches to the assessment and treatment of myriad complex medical conditions (25, 26).

Systems thinking has a long history in psychiatry, going back to the development of the notion of homeostatic regulation of physiological systems in the work of Walter Cannon (27), and some of the early applications of systems theory (28). Psychiatrists and neuroscientists were key figures in the development of cybernetics in the 1940s and 50s (29). This work aimed to model learning and adaptation in mechanistic terms and identify forms of pathology with specific types of dysregulation of adaptive systems. Subsequent work applying systems thinking to understand psychopathology was inspired by systems biology (32), the cybernetics of behavioral control systems (33–36), complexity theory (37, 38), and recognition of the impact of social-structural determinants of health (39). A recent version of control systems modelling can be found in the active inference approach to explaining specific forms of psychopathology (40). While focused initially on neural processing, active inference can be readily extended to consider interactions with the environment and social networks, (41–44).

Systems theory and cybernetics were central to the development of family therapy (45–48). Families were viewed as self-regulating systems comprised of individuals in interaction with each other (49). These interactions are influenced by individuals’ characteristics but also reflect spatial, material and symbolic structures as well as the social practices, norms, rules, and rituals that constitute family life. The family system is constituted both by the individuals who are its members and the community, society or culture that configures and constrains its structure and identity. The family system thus serves the needs of its members and of the larger society in which it is embedded—and these diverse needs may sometimes conflict with each other. While there have been substantial efforts to elaborate sets of dimensions, typologies, and measures to characterize the structure and dynamics of families [e.g., (50–54)], to date, none have achieved wide acceptance or clinical application. The interactional view of family systems has been extended to consider larger social networks and structures (55-58) but this is also an unfinished project (59).

In the 1970s, second-order cybernetics theory emphasized the role of recursivity, self-reference and self-construction (*autopoiesis*) in living systems (60). This opened the way to a deeper engagement with social, cultural and linguistic processes of meaning making (39, 61–63). Agency, subjectivity, and narrativity are given central place in systems approaches that acknowledge the role of communication, storytelling and self-reflection in human experience (45).

There are many interconnections among these different approaches to systemic thinking in psychiatry. This reflects both their shared genealogy‚ with common ancestors, and cross-fertilization among disparate strands. These lines of work are complementary and each can address some of the limitations of the others. In particular, systems neurobiology focuses on multilevel processes in the nervous system but does not sufficiently consider the social environment or treats it simply as a modulator of neural processes rather than as constitutive of brain function. Systems biology has been extended to consider biosocial interactions, but this work often does not specify the psychological processes of meaning and experience that mediate bodily and social interactions (64). Krieger’s (65, 66) ecosocial epidemiology uses the construct of embodiment to emphasize the biological effects of structural inequities (i.e., how adversity gets “under the skin”) but also does not clarify psychological processes. Bronfenbrenner’s (67-68), bioecological psychology emphasizes the dependence of developmental processes on environmental context but its application has not always considered the interaction of the multiple levels of social organization in which individuals and families are embedded (69). Ecocultural approaches grounded in ethnographic methods have provided ways to characterize the culturally constructed meanings and practices that constitute lifeworlds and developmental pathways (70). The notion of an ecology of mind, introduced by Bateson (61, 71) views cognition as emergent in loops of individuals interacting with the environment and through interpersonal communication with other humans in a social system (72). The many strands of 4E cognitive science develop this perspective in terms of processes of embodiment and enactment that involve social embedding and extension in the world (73–76). The cultural psychiatric perspective emphasizes the interactions of individual and collective meaning making and the social-political contexts of institutional power and practice that create cultural niches and affordances (77). Computational methods allow us to put aspects of each of these approaches together in an overarching model that can reveal system dynamics (78). The novel aspects of our approach that distinguish our framework from previous work include: the explicit integration of culture (as embodied background knowledge and enacted situated practice); the characterization of basic psychological processes of subjectivity, narrativity, and agency in terms of embodiment and enactment; and a focus on the dynamics of multi-level biological, cognitive and sociocultural looping effects as potential mechanisms of pathology and targets for intervention.

## Hierarchical systems theory in biology

Biological systems are hierarchically organized, with components that are arranged in ways that give rise to stable structures with new properties and processes (20). For example, the metabolic processes of the cell depend on the spatial organization of enzymes on its membranes. The computational functions of the brain depend on its hierarchical structure of networks and nodes (79). This organizational process is recursive and new control processes emerge as a result of the hierarchy (34). This hierarchy includes the social environment which emerges as part of specific arrangements of relationships with others through social norms, rituals, institutions, and practices—and which, in turn, shapes the development and functioning of the individual.

The notion of hierarchy sometimes conjures images of domination or oppression. However, as we use it here, hierarchy does not involve value judgments about degree of importance, power or privilege but refers to specific forms of organization of systems (80). Some philosophers are critical of the idea of hierarchy and levels in living systems because they see this as imposing a misleading model or metaphor on phenomena that are fluid, shifting, or ‘holistic’ (81).^1^ Others are concerned about the notion of ‘top-down’ causation, arguing that causal processes can only involve same-level processes that are materially linked (83). In reality, top-down causation is common in complex systems and is central to organismic biology (84). There are many types of organization that can be discerned in the world or applied to experience and the utility of concepts of hierarchy and levels depends on the specific question, problem, object of interest and pragmatic task at hand (85, 86).^2^

The notion of hierarchy is used in multiple ways in biology that include *subsumption*, *composition*, *scale*, *causality*, and *control* (88, 89). Hierarchy implies organization into levels, but the significance of these levels differs in each of these versions of hierarchy. In biology, each of these notions of hierarchy is useful but the one that is most important for an ecosocial systems view in psychiatry is that of control hierarchies.

*Subsumption hierarchies* are classifications in which something is seen as a member of instance of a larger category. An example is a Linnean taxonomy of species taxa. The logical relationship between levels can be captured by set theory. The elements of progressive levels are sets of the prior level’s sets. Elements at lower level may be viewed as concrete instances, while higher levels are abstractions, or each level may have a kind of ontological identity (90). A lower-level instance can stand metonymically for the whole. But the way that elements are grouped into larger sets of sets can provide a conceptual structure, represented by a graph or lattice that represents the way that the groupings are based on specific facets or properties of the elements.

*Scale* refers to the number or the size of the assembly relative to its components, spatial or temporal span. Scale differences can be continuous or discrete. Some biological and social network-based phenomena are ‘scale free’; that is, the same structural organization and dynamics are observed at multiple scales or else scale-up in a quantitatively predictable way (91, 92, 93). This allows dynamical system models to be applied in an iterative way to characterize processes across these networks at multiple scales. However, many physical and biological phenomena are not scale free; that is, size matters(94–96). The sheer number of elements, their topological arrangement or connectivity, and their spatial or temporal extent can give rise to new dynamics. In this case, the emergence of new dynamics marks a new level in a hierarchical structure.

In *compositional hierarchies*, the focus is on part-whole relationships (97). The parts are building blocks that are arranged in spatiotemporal structures that create a new level of organization. Bricks are laid to build a wall; walls joined to build a room; rooms are concatenated to build a house; houses are arranged to create a neighborhood. The process of composition may involve different kinds of arrangement at each level and similar processes may be involved in stabilizing the structures (e.g., mortar may be used to build walls, to join them into rooms, and to join rooms into a house). However, different processes (reflecting other properties of the components or additional components) may stabilize structures at different levels (buildings might be joined by mortar to build a wall and walls might be joined by interleaving bricks at a corner, or by at angle brackets made of metal). Depending on our focus of study, the level and processes we need to explain a phenomenon will shift. Thus, if we are looking at the stability of a house we will be interested in the strength of bricks and mortar bonds, and the buckling properties of columns and frames; whereas, if we are interested in neighborhood stability, we will need to consider parameters at other compositional levels like street layout, greenspace, and social relationships among inhabitants. However, we may find that house stability and neighborhood stability significantly affect each other because of mechanisms that link these through social and economic processes such as house pricing, gentrification, neighborhood pride and upkeep.

Compositional hierarchical organization is central to biology and essential to phylogeny, ontogeny, and adaptation to new environments because biological systems build on existing structures by preserving, re-organizing, and re-purposing components (98). In biology, there are multiple compositional hierarchies, but the main line follows from the ways that processes are stabilized to create a hierarchy of material structures (99): molecules are joined to make macromolecules (through chemical bonds); macromolecules are arranged in space (with the aid of membranes and other macromolecules) to produce organelles; organelles are arranged in space (again with the aid of membranes, macromolecules and other organelles) to create cells which have metabolic cycles; cells are organized into tissues which have biomechanical and other functional properties; tissues are organized in organs which can perform multiple functions related to their structure and anatomical location; organs form physiological systems, which have properties related to interactions between the organs they connect; physiological systems constitute organisms; organisms form communities; and diverse communities in environmental context constitute ecosystems.

*Causal hierarchies* reflect arrangements determined by mechanisms or processes that produce a given effect (100, 101). The directionality of the link (or irreversibility of the process) establishes an ordering. The ordering of causes leading to outcomes which are causes of subsequent outcomes provides a sequential structure that can be described as a chain of cause and effect. Of course, most processes have multiple causal contributors that interact and result in different partial orderings or lattice structures that may have a layered or hierarchical structure. Moreover, multiple causes may independently lead to the same outcome (*equifinality*), and single causes may lead to multiple outcomes (*multifinality*), presumably reflecting the influence of other historical or concurrent causal factors. Finally, the assumption of unidirectionality at one causal level may not hold when the larger system of relationships is considered. Most biological systems involve mutual or circular causality or feedback loops. Indeed, circularity (autocatalysis, self-assembly or autopoiesis) is essential to what characterizes a system as living (102–106). Through such circularity and self-reference, biological systems then instantiate another form of hierarchy that involves self-regulation or control.

*Control hierarchies* are defined in terms of successive levels of regulatory loops (107). The control systems perspective is especially relevant to understanding biological processes (and psychopathology) because it leads to a useful way of understanding function and dysfunction in terms of the goal-oriented nature of behavior and adaptation. A basic building block is a feedback loop in which a state of the organism or environment is compared with an expected (or desired) state [what Miller et al. (34) called a ‘Test-Operate-Test-Exit’ or TOTE unit]; the discrepancy then drives a compensatory action (either revising the expectation or acting on the world to make it better conform to the expectation). Successive levels are loops of loops. These loops can involve different processes that are best characterized as regulating information (or ‘free energy’) rather than energy *per se* (108). This is the kind of hierarchy of greatest interest in making sense of the dynamics of living systems. For living systems, these loops are characterized by a fundamental regulatory goal of maintaining organism integrity and persistence in the service of reproduction and other goals. The resultant *teleodynamics* distinguish living systems from other regulatory systems that lack the capacity to generate organism-specific goals and norms and to function in ways that are explicitly informed by future possibility (109, 110).^3^ In humans, this process extends to the self-reflexive, imaginative and cooperative processes of agency enabled by language and culture (112, 113).

The general idea of hierarchy then *does not* imply unidirectional (top-down or bottom-up) causation, linear dynamics, or reductionism. In fact, evidence for hierarchical organization is seen in many emergent phenomena. Emergence involves the appearance of new levels of organizational structure without implying loss of underlying structures or component levels (114). These new levels of organization have their own dynamic processes. The emergence of new structures with distinctive properties and of processes with new dynamics warrants the use of the concept of *levels of organization* and corresponding levels of description.

In hierarchical systems, the function of each level can be explained not only through the interactions of its components but in terms of its relationship with both higher and lower levels. For example, the genome is a set of structures used by the cell to regulate its activity and replicate itself; the genome itself is a dynamic system that is regulated by a network of macromolecules (115). Similarly, the cells of a healthy multicellular organism serve the priorities and plans of the whole organism—sometimes to the detriment of their individual survival.^4^ The functions of any level in a biological system then only make sense in relation to the dynamics of the larger system, including the regulatory processes organized at higher levels. The principle of *biological relativity,* developed by physiologist Denis Noble (117–119), argues that in biological systems causal chains can begin anywhere within the system or hierarchy. This approach to systems biology recognizes the organizational value of hierarchy but is explicitly anti-reductionist in the sense that both lower and higher levels of organization have causal efficacy and contribute to the dynamics of the system as a whole or the subsystems that constitute brains, persons, families and communities.

## Multilevel explanation In psychiatry

The biopsychosocial (BPS) approach championed by Engel (120, 121) promised a conceptual framework to integrate multiple levels of analysis in psychiatry based on general systems theory (12). The motivation for this was a concern to give a place in clinical theory and practice to the intrapsychic processes characterized by psychodynamic theory and patients’ own experience and understanding of their condition (122). But the definition and operationalization of these level and their exact interplay in cross-level formulations, were left undetermined. Critics of the BPS, like Ghaemi (123) have argued that the framework is little more than a placeholder with no real content to guide diagnostic assessment, formulation and treatment (124). To a large extent, this claim says more about critics’ failure to engage the burgeoning literatures of systems biology, psychophysiology, family systems theory, social epidemiology, and other social sciences, which can put ample flesh on the bones of the BPS model, than about any inherent limitations of a multilevel systems approach to health and illness (125, 126. The lack of engagement with this literature is evident in Ghaemi’s alternative proposal that psychiatry employ mechanistic biological accounts of disorder complemented by phenomenology and a humanistic concern for patients’ experience. In this approach, the causal mechanisms of psychopathology are divorced from the social world. Subjectivity and social context are acknowledged as important to ensure a humane engagement with the patient but are not seen as primary mechanisms of pathology and are taken for granted as aspects of the patient’s clinical presentation that can be adequately accessed and assessed with empathy and common sense.

While Ghaemi’s concern that the BPS leads to “undisciplined eclecticism” seems to us to be unfounded, more valid concerns are that in practice the BPS remains mainly descriptive rather than dynamic, simply enumerating potential risk, causal or maintaining factors, without detailing causal mechanisms that could guide intervention. Perhaps this is why, despite its widespread acceptance, the BPS has failed to prevent or reverse the adoption of reductive biological explanations in psychiatry. Moreover, while the BPS was motivated by concerns to include patients’ lived experience, even mental health practitioners who claim to use a BPS approach tend to neglect subjectivity and social-cultural context. This failure may reflect the lack of interdisciplinary training (127,128), the difficulties of conceptual integration (81), and the persistence of dualistic thinking (129).

We start from a different premise, supported by a wealth of research in psychosomatics and sociosomatics, that insists that symptoms and syndromes in psychiatry arise from the interaction of psychophysiological, cognitive-affective, and sociocultural processes (77). Psychiatric disorders are complex, multidimensional constructs, and symptoms are more than just indices of an underlying neurobiological mechanism that can be captured by biomarkers (130, 131). Psychiatric disorders emerge within loops that involve the biology of human adaptation as well as cultural practices of diagnostic labelling, health care systems and larger discursive formations. Illness experience therefore does not follow directly from pathobiology but is embedded in cognitive and social processes that mediate and modulate the translation of physiological or psychological disturbance into symptoms and behaviors. This transduction and translation occurs at multiple levels that involve symptom schemas and their interaction, interpersonal responses, narrative conventions, social positioning, the health care system, economic constraints and sociopolitical processes (132)^5^.

This perspective is consistent with recent work in symptom network theory, which suggests that psychiatric disorders result from the dynamic interaction of multiple symptoms each of which may have its own pathophysiology or psychopathology (133), (136). Instead of assuming that a single latent construct can explain the symptom patterns that characterize psychiatric disorders, network analysis views disorders as systems of causally connected symptoms (137). These causal connections can involve physiology, behavior, experience and interpersonal interaction, as well as the responses of social institutions and the environment. While some authors consider a network as an inherently non-hierarchical structure, causal or control hierarchies may be part of the mechanisms that constitute and connect symptom networks, not as a matter of composition (or latent constructs) but as part of causal chains or loops. The ecosocial systems view we outline in this paper extends the idea of symptom networks to include social-cultural contexts, self-reflection and narration as active causal processes (1).

These multiple levels of process reflect structures that are organized hierarchically in the sense that higher organizational levels involve arrangements of structures at lower levels that give rise to new processes that require new conceptual vocabularies to describe. For example, the brain is composed of functional circuits, which are made up of neurons; the social world is made up of roles, niches and institutions which are constituted by patterned relationships among individuals, whose behavior is regulated by cognitive maps, models and affordances, social positionality, norms, and conventions (138). Each level enables processes that contribute to the causal mechanisms that underlie a particular symptom, syndrome or affliction (139). Experience, behavior, narrative self-understanding, and social interactions can all contribute causally to the dynamics of psychiatric symptoms and disorders (140–142).

Even brain-based explanations of mental disorders require an appeal to multilevel systems dynamics (143). Changes in synaptic function or neural circuitry alter information processing, which in turn gives rise to changes in social behavior and experience(144). The process is bidirectional. Psychotherapy and other psychological interventions have effects on the brain(145). Changes in social behavior alter brain function in ways that may be self-sustaining or create knock-on problems in other brain systems or behavioral functions. Social environments and models of the self in context influence neurobiology, immunology and inflammatory processes (142).

Beyond neurobiology, mental disorders also involve cognitive, affective and attentional processes that emerge from particular learning histories and narrative modes of recollection and self-narration, as well as interpersonal interactions with others in one’s family, community and wider social networks. These social interactions have their own dynamics that may aggravate or mitigate symptoms or create predicaments that present their own challenges to health and well-being. Social interactions can also feed back into cognitive and bodily processes in ways that amplify or diminish symptoms and distress. These loops correspond to relationships between different aspects of the organism or between the organism and the environment. Loops may result in cycles of positive and negative feedback, with effects locally as well as across the organizational hierarchy. Depending on their structure, parameters and initial conditions, loops can result in nonlinear dynamics, for example, growing exponentially, showing discontinuities, bifurcations, or other complex dynamics (146, 147). To the extent that these loops have their own dynamics, they can be viewed as specific mechanisms that need to be considered in diagnostic assessment and case formulations and that can be the target of clinical intervention. Moreover, because human adaptive systems involve regulatory or allostatic processes with specific goals or set points, they may exhibit *equifinality*, in which, despite variations in initial conditions and ongoing perturbations, they tend to follow a predictable trajectory.

Identifying these stable patterns or trajectories could provide a basis for a typology of disorders organized in terms of regulatory processes that exhibit stable attractors, limit cycles, and final common pathways.^6^ If these can be identified and empirically validated, they could be used as a basis for diagnoses that are *prognostic* (predicting outcomes) or that indicate potentially effective treatments, and that point to specific targets for intervention. This systems-based nosology, however, will generally be quite different than simply identifying single mechanisms, causal factors, or etiologies for disorders because it involves dynamic properties of systems with looping effects.

A typology of looping effects (vicious or virtuous) could complement current diagnostic nosology (149). This enlargement of frameworks would not completely supplant current nosology, which has its uses, insofar as it captures salient aspects of illness experience and can be related to prognosis or differential therapeutics. Clinical assessment routinely goes beyond diagnosis to include a problem list—some categories of which are included in the ICD and DSM-5 Z-Codes (150, 151)—and case formulation that may note contextual factors, but this process is unsystematic. Efforts to systematize the inclusion of social context and determinants of health in assessment are urgently needed. This needs to go beyond a laundry-list of factors to include dynamics. Person-centered diagnostic assessment includes characterizing strengths and resources, risk and protective factors, and relevant developmental, ecological and meaning-centred contexts (152). Attention to looping effects could be incorporated into current practice through case formulation and systemic intervention without waiting for the development of a systematic nosology. Table 1 lists some of these potential loops both within levels or domains and across levels using depression as an example.

**Table 1:** Examples of Looping Effects Related to the Mechanisms of Depression and Treatment Response.

Domains	System dynamics and looping effects		References
	Within levels	Across-levels	
**Neurobiological**	Psychopathology involves self-sustaining loops in neurobiological, autonomic, endocrine, and other regulatory systems that are related to reduced stress tolerance and increased vulnerability to chronic stressors	Depression is linked to HPA dysregulation which leads to impaired stress response, and to symptoms including alterations in sleep, appetite, reward processing, emotion regulation and cognition. These alterations affect cognition, coping and interpersonal interactions in ways that can exacerbate depression	(153) (154)
	Treatments that alter synaptic transmission lead to habituation or compensatory responses; this might decrease the efficacy of some medications over time, cause rebound on medication cessation, and increase the risk of relapse; e.g., denervation supersensitivity from receptor blockade	Decreased efficacy of medication leads to fear of relapse, demoralization, decreased self-efficacy, social avoidance, and, ultimately, less efficacy of medication Rebound effects of medication contribute to more challenging withdrawal and continuation of medication	(155)
	Medication works at multiple brain and body sites and affects systems with multiple functions causing ‘side-effects’ that may contribute to or undermine therapeutic efficacy	SSRIs can reduce emotional reactivity with impacts on emotional responsiveness, self-understanding and ability to connect to others. SSRIs interfere with sexual function and decrease libido, which may have negative effects on self-esteem and on intimate relationships	(156) (157)
**Psychological**			
Affective	Impaired emotion regulation leads to decreased cognitive flexibility, increased irritability, dysphoria, anxiety with consequences on sleep, cognitive processing including negative bias and self-appraisal, worry and rumination, problems with impacts on learning and performance which reduces stress tolerance and increases emotional distress	Impaired emotional regulation has negative impacts on goal-directed behavior and can increase perceived chronic stress which, in turn, is linked to HPA dysregulation and maladaptive coping (e.g., dysfunctional behaviors such as substance use and social withdrawal)	(158) (159) (160)
	*Emotional distress* interferes with functioning, leading to performance decrements, negative self-appraisal, and greater emotional distress	Emotional distress is linked to others’ response to emotional expression and can lead to interpersonal problems and avoidance of social situations with loss of social support, and increased experience of loneliness	(161) (162)
	Mood influences memory, leading to difficulty accessing mood-incongruent memories, and greater recollection of mood congruent memories, reinforcing dysphoric mood	Depression alters autobiographical memory, which leads to negative self-presentation, impaired social functioning and more negative memories	(163)
Attentional	Attention to negative social cues increases sense of threat and difficulty in social functioning Reduced attention to positive stimuli	Increased attention to negative social cues and signs of failure exacerbates depressive mood and social withdrawal; Focusing on positive faces reduces dysphoria	(164) (160)
Attributional	Attributing sensations to pathology leads to the conviction that one is ill, increasing the tendency to attribute sensations to pathology	Attributing sensations to depression leads to depressed mood	(165)
Embodied experience	Bodily habitus, stance and facial expression shape experience	Slumped posture, frown influence feelings of depression	(166), (167)
**Social - Micro**			
Family systems	Family influences development across the lifespan and also provides a niche and resource for adaptation	Early adverse experiences both *in utero* and in early childhood can initiate changes to basal and stress-related cortisol secretion. This impacts stress tolerance. Caregiver response in infancy shapes interoception, self-regulation, ability to attune and attach, also laying the ground for future interpersonal relationships and response to perceived stress. Depression alters family dynamics	(168)
Interpersonal	Reactions of others to distress influences illness experience and coping Withdrawal of others leads to emotional distress and behaviors that prompt further withdrawal by others	Social withdrawal can lead to lack of perspective fostering feedback and support which may lead to deepening of dysfunctional behaviors and negative self-biases in addition to limiting corrective experiences. Depressive symptoms lead others to increase social distance Social rejection alters neural functioning in ways that can lead to further withdrawal Behavioral activation leads to increased social activity with more rewarding experiences improving mood leading to greater activity	(169) (170)
**Social - Meso**			
Neighborhood	Neighborhood and community can modulate impacts of micro and macro-level factors Sense of belonging and access to a social network/community contributes to wellbeing and social capital with impact on opportunities to thrive	Sense of belonging and support impacts sense of agency and self-identity. Experiences of being excluded, judged or ostracized as part of a community can lead to social withdrawal or isolation, self-doubts, loneliness and induce other dysfunctional behaviors and impair coping	(171) (168)
Work	Job loss impacts self-esteem, social standing, resulting in low mood, and economic hardship	Low mood and demoralization impede job search, performance and retention Others response to job-loss can shape coping strategies and amplify distress	(172)
Health care system	Type and availability of health care services and caregiving increases the tendency to seek care for specific types of symptoms or concerns	Distress is shaped by diagnostic categories and available treatments. Treatment response (which may include placebo effects) validates diagnostic categories	(173) (174)
**Social - Macro**			
Economic	Poverty increases risk for depression Financial stress can lead to negative affect and dysfunctional behaviors that worsen economic adversity	Depression increases risk of poverty Poor cognitive performance can impact economic status including status, reputation as well as income and assets.	(175) (176)
Transnational	Marketing of pharmaceuticals influences the availability of specific diagnostic labels and treatments, which are applied to patients who then become consumers of medications, increasing economic demand and encouraging further marketing	Reliance on medications increase sense of vulnerability and impairs coping May also impact agency and identity development	(177) (178)

Although loops are difficulty to study, they are composed of causal arcs that can be characterized with existing methodologies. Table 1 lists many such causal arcs that linked together would result in ‘loopy’ dynamics. This kind of model is central to cognitive theories of depression and anxiety (179), which have led to effective treatment interventions and can readily incorporate cultural-contextual factors (180). There have been some notable successes in identifying predictors of dynamics in couple interactions (181). New experimental methods have been developed to study the dynamics of dyadic, family, and group interactions (182–184). Symptom network theory and computational modelling provide new approaches to examining looping dynamics, testing the relative strength of specific linkages and the sensitivity of network dynamics to changes in parameters that can be matched with measurable variables in research and clinical applications [e.g., (185–187)]. In clinical settings, nonlinear dynamics are commonly observed and putative explanations in terms of loops could be tested by interventions that target specific parameters (188, 189).

Identifying the feedback loops that may contribute to psychopathology is difficult. Statistical methods can be used to show time-lagged autocorrelations and cross-correlations in observational data that suggest feedback dynamics (190, 191). Experimental methods that manipulate particular parameters or control the nature of physiological, perceptual or interpersonal feedback can provide firmer evidence for feedback mechanisms(192). Computational models can be constructed that capture some of the interactions and identify parameters that affect dynamics (193). However, in practice, these usually are simplified ‘toy’ models that do not include many of the loops and variables present in real-world contexts. This may lead to mistaken predictions or over-generalization. There is a need for an extensive research program of modelling built on large datasets that include potentially important individual and contextual variables (194).

Applying computational models in clinical settings poses additional challenges related to the constraints of clinical epistemology. The data available for an individual patient may be very limited and not include a time-span necessary to reveal dynamics. The interventions that clinicians make are not really single-subject experiments because they occur within a context of expectations and demands that heavily constrain patients’ response. The patient’s own interpretations and self-construals affect the impact of any intervention and any subsequent interaction with the clinician. Hence, we need a circular hermeneutics to complement our models of circular causality (195). The system of patient and clinician must be included in the model and situated within the larger ecology of health care and adaptation in social context.

Crucially, the loops relevant to clinical concerns include modes of self-construal based on cognitive, social and cultural models, institutions and practices (77). For example, the interpretation of experiences of pain, fatigue or lack of interest as symptoms of depression is a culturally shaped attributional process that leads to particular modes of coping and help-seeking (149). These attributions may be re-negotiated in clinical and other social contexts with others who may validate or contest the views of patient or physician (196). To the extent these social and clinical responses validate the individual’s self-construal, they constitute a loop in which the available categories for symptom interpretation and clinical practices reinforce each other—an instance of what Hacking (197) has called “the looping effect of human kinds.” These loops may be internal to the individual, involving bodily attention, interoception, and physiology (examples of what Hacking (198) termed “biolooping”) or they may primarily involve cognitive and social-rhetorical processes that reconfigure the sense of self (173, 199). Loops also may be irreducibly social or political, changing the larger environment and available narratives in which social position and structural adversity determine the causes and course of symptoms. Psychiatry itself as a social institution participates in these loops through diagnostic labelling, discursive practices, and modes of social control that may aggravate or ameliorate suffering (200, 201). The types of problems included within the purview of psychiatry, the kinds of explanation and interventions used, and the larger context of practice are all part of the dynamic system that shapes experience and behavior.

## 4E cognitive science as a path to multilevel integration

Contemporary 4E cognitive science points to ways to conceptually integrate multiple dynamic levels of organizational complexity that involve neurobiological, social, cultural, and environmental contexts across spatio-temporal scales (187, 202–205). The 4E cognitive science approach argues that cognitive processes are *embodied*, *embedded* in social contexts, and involve *enactments* that *extend* into the world. *Embodiment* refers to the ways in which the body provides a scaffolding for cognition and experience.^7^ *Enactment* emphasizes that embodied experience emerges through ongoing cycles of action and perception that engage the environment. Cognition serves adaptation, and a changing environment requires action to maintain the body and the person in a healthy, functional state (208). Human adaptive niches are cooperatively constructed. Action and experience therefore are *embedded* in social-cultural contexts. The action-perception cycles of cognition *extend* beyond the body to engage with the material and cultural affordances of a local niche and larger social systems. From a 4E perspective, both the experience and the mechanisms of health and mental disorders can be approached in terms of individuals’ dynamic engagement with the social world.

Dynamic engagement with the social world requires constant adaptation and resource optimization. The concept of *allostasis*, which refers to the ways in which organisms anticipate and adapt to challenges, focuses on the function of physiological and biobehavioral systems of stress response and regulation (208, 209). Allostasis involves the organism’s capacity to allocate resources to maintain an adaptive balance between coping and recovery in response to adverse conditions and events. This involves both internal physiological processes and behavioral strategies based on appraisal of challenges and available resources for coping (210). When allostatic regulation is insufficient, various forms of stress-related dysfunction can result from has been described as ‘allostatic overload’ (211).

The processes involved in allostatic regulation can be viewed from an enactive perspective as ongoing cycles of action-perception (212). They can also be modelled as Bayesian processes of active inference, in which the organism predicts and acts on the environment to ensure its own stability (108). These cycles occur internally through interoception and physiological regulation of the internal milieu and externally through behaviors that act on the body and the environment (213, 214). Cycles of action-perception also underlie our sense of agency both in terms of the sense of volition and control (215), and the wider sense of being able to change our social circumstances (216-218). The action-perception cycles that are constitutive of agency and subjectivity emerge in and are maintained by social-cultural contexts that involve other people in dyads or couples, families, neighborhoods and communities, as well as larger social networks and institutions (219). These larger ecological domains contribute to higher-order goals and plans. Problems in self-regulation and adaptation can originate at any level in this system, with potential repercussions throughout. Hierarchical organization of goals is part of healthy functioning and certain forms of psychopathology may result when stress or allostatic overload disrupts this organization (220).

Healing practices, therapies and treatment interventions can work to restore allostatic function where it has been disrupted. The overall aim of allostasis is to adjust regulatory systems to maintain the health, survival and reproductive fitness of the individual. More proximally, this includes responding to the challenges and demands of a social niche in ways that fit local cultural norms, roles and expectations. This may involve changing perceptions (learning new ways to attend to and interpret sensations from the body or the environment), taking new actions (enlarging the repertoire of behaviors and changing plans and priorities), or re-establishing links between action and perception that have been disconnected (providing feedback from outcomes that can guide recursive goal setting). Both internal changes and actions on the world can participate in the same adaptive cycles.

The 4E approach can be readily extended to include the essential functions of language in human adaptation (221). Humans are language animals (113), inhabiting a world that is comprised not only of physical arrangements but saturated with linguistically mediated meanings, which provide the content of social norms and conventions as well as the scaffolding for the construction of a narrative self. The *narrative practice hypothesis* focuses on how this linguistic capacity emerges developmentally through culturally prescribed practices of self-narration, giving rise to folk psychology with its grammar of motives, plans and intentions that are employed to organize memory and action, articulate individual goals, and offered to others as reasons and explanations for one’s behavior (222). Linguistic capacities allow regulation of systems that are organized in terms of physical dynamics because narrative construals of self and context organize, constrain and modify lower-level action plans both within individual cognition and in communicative interactions with others. Language is self-referential and recursive and, through metaphor and narrative, is used by individuals and groups to construct novel multilevel hierarchies that regulate complex cognition and behavior. This is a key facet of the ways that culture permeates human cognition and functioning. Of course, language and culture reach deeper to reshape cognition, perception and action in ways that are nonconscious, implicit and automatic (167, 207, 223, 224).

Throughout the lifespan, culture shapes the human nervous system, allowing us to navigate socially constructed environments, engage in cooperative activities, and pursue our goals through embodied knowledge, skills, habits and dispositions (225). But much of culture remains outside the individual, distributed among others with specific expertise, residing in relationships, reproduced in institutions or practices, and present in social niches that provide cultural affordances for action and perception (226). These cultural affordances are part of the extended context on which human cognition and adaptation depend. Central to this context are interactions with other people, texts, and institutions. We rely on these interactions in local niches and relationships or larger networks to scaffold cognition, guide behavior and augment our capacities by “thinking through other minds”—whether in ongoing cooperative interactions with others or by consulting the vast archives of human knowledge and experience (44).

In summary, current elaborations of 4E cognitive science offer an account of human function in dynamical systems terms as embodied (coupling bodily physiology and experience), enacted (involving sensorimotor loops that give rise to agency), embedded (context sensitive), and extended into the environment (dependent on cultural affordances). By tracking the ways that processes of organismic self-regulation and experiential learning emerge from ongoing cycles of interaction between the individual and the social-cultural environment, this framework can integrate physiology, cognitive processes, including individual agency and self-construal, and participation in cooperative meaning-making. This allows us to recast basic processes of symptom production, distress, coping and adaptation as well as the response to interventions in terms of multilevel dynamical systems. This systemic view opens the way toward a conceptual approach that considers how the co-constituted systems of body, mind and person are in transaction with larger interpersonal, social and cultural systems.

## Integrative case formulation

Comprehensive diagnosis and treatment in psychiatry requires addressing pathology in all its dimensions: biological, psychological, social, cultural, and environmental. Integrating these into causal explanations of particular types of problems remains a challenge for psychiatric theory and practice (126, 227). Approaching these multiple forms of explanations as independent or even incommensurable ignores the obvious ways in which processes at multiple levels not only affect but mediate each other. An ecosystemic approach to integration aims to identify multiple causal processes or mechanisms within and between levels of organization and articulate their connections in an overarching system.

Advancing integrative case formulation requires approaching the patient as embodied and embedded in an ecosocial niche that presents an array of inter-related social determinants of health with differential constraining and enabling opportunities. The same niche also provides models for self-understanding, values, aspirations, and afflictions that shape experience, adaptation, coping, and help-seeking behavior, as well as access to services, educational and vocational opportunities, and other resources. Individuals’ responses to adversity, symptoms or disorders, and modes of recovery will be influenced by the norms, expectations, and constraints of the sociocultural contexts they inhabit.

To illustrate how this integrative perspective works in clinical practice, consider the following case vignette^8^:

A 30-year-old woman presents to a mental health clinic with a self-diagnosis of depression. On inquiry, she reports feelings of emptiness, worthlessness, and guilt, as well as irritability, restlessness, rumination, difficulty concentrating, indecisiveness, early awakening, and fatigue over the past 6 months. Most recently, she has had increasing loss of interest and pleasure in ordinary activities and social isolation, as well as thoughts of death. She has done some online research and comes to the clinic asking for laboratory tests to confirm her diagnosis and determine the best treatment. She recently read a blog that mentioned novel research findings on the use of brain imaging and pharmacogenetics in personalized treatment for depression and presents the clinician with a list of private labs that offer this service. On further discussion, she reports that she lost her job three months ago and feels deep humiliation. She also mentions having difficulties in her relationship with her partner, saying that they are “going through a rough patch.” She explains that she feels anxious and out of control and at times fears that she is “losing my mind.” She is prescribed an SSRI antidepressant and experiences some lessening of her symptoms over the next few weeks, but does not feel any return of sexual interest, which adds to her worries about her relationship.

As is increasingly common in mental health care, the person in the vignette presents clinically with a self-diagnosis of depression and, in this case, expects treatment with medication for what she views as a brain-based disorder. She also has ongoing social stressors that may be both causes and consequences of her mental state. How she interprets her symptoms and her feelings of anxiety, hopelessness, humiliation, guilt or shame will affect both her behavioral and neurophysiological response to the predicaments of job loss and relationship strain. In addition to temperamental traits or constitutional predispositions and the neurobiology of mood regulation (228), a complex interaction of embodied processes—shaped by previous illness experience, life events, and the response of others—add reinforcing or attenuating loops that further complicate the system dynamics that underlie symptoms and distress. A clinically effective approach to explain and treat distress therefore must go beyond neural correlates and biomarkers to consider individual variations in phenomenology and lived experience (229, 230), developmental processes (231, 232), symptom trajectories (233, 234), and socio-cultural dynamics, which depend on social structure, institutions and practices, as well as cultural systems of meaning (218, 235, 236).

**Figure 1:**
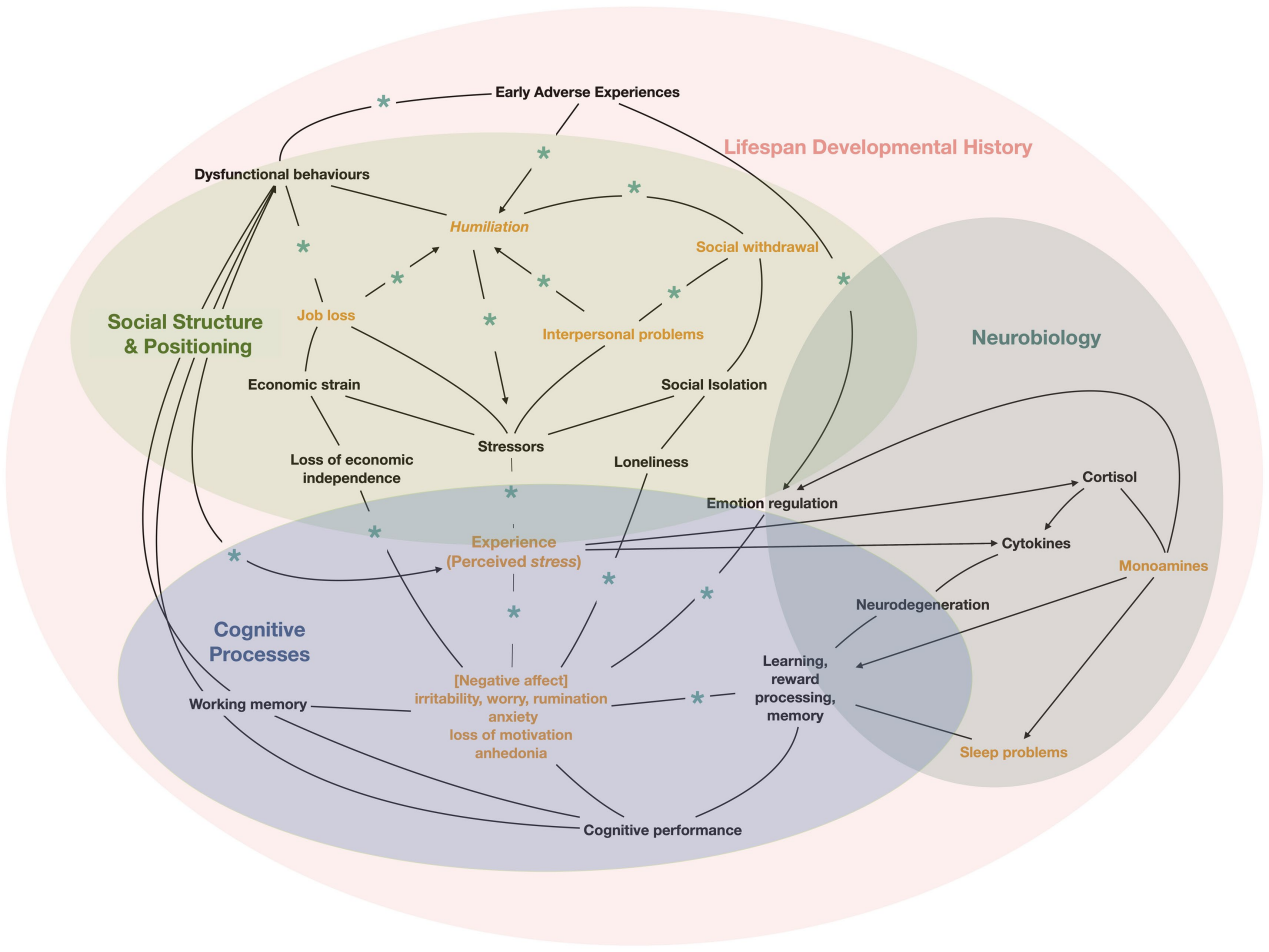
Ecosystemic Embedding of Depressive Symptoms. The figure illustrates some of the many links between symptoms, processes and experience that constitute the ecosocial system of the patient described in the vignette. The arrows represent causal influences mediated by diverse mechanisms. Closed loops can give rise to feedback amplification, resulting in vicious cycles of symptom exacerbation or, when regulatory mechanisms are sufficient, can lead to allostatic changes that contribute to resilience and recovery. For the links marked with asterisks, the mechanisms of influence depend on nonverbal and linguistic communication through embodied and enactive loops that give rise to intersubjectivity, positionality and ongoing negotiations of meaning as depicted in Figure 2. Based in part on (237).

In the case of the patient in the vignette, the causal mechanisms of anxiety, demoralization and depression can (and likely do) start at many different points in the network depicted in Figure 1. Additionally, each of these processes can interact with potentially reinforcing or compensatory feedback loops. These dynamics are important for adequately characterizing the nature of the problem, its likely course or prognosis, potential interventions, and treatment response.

**Figure 2:**
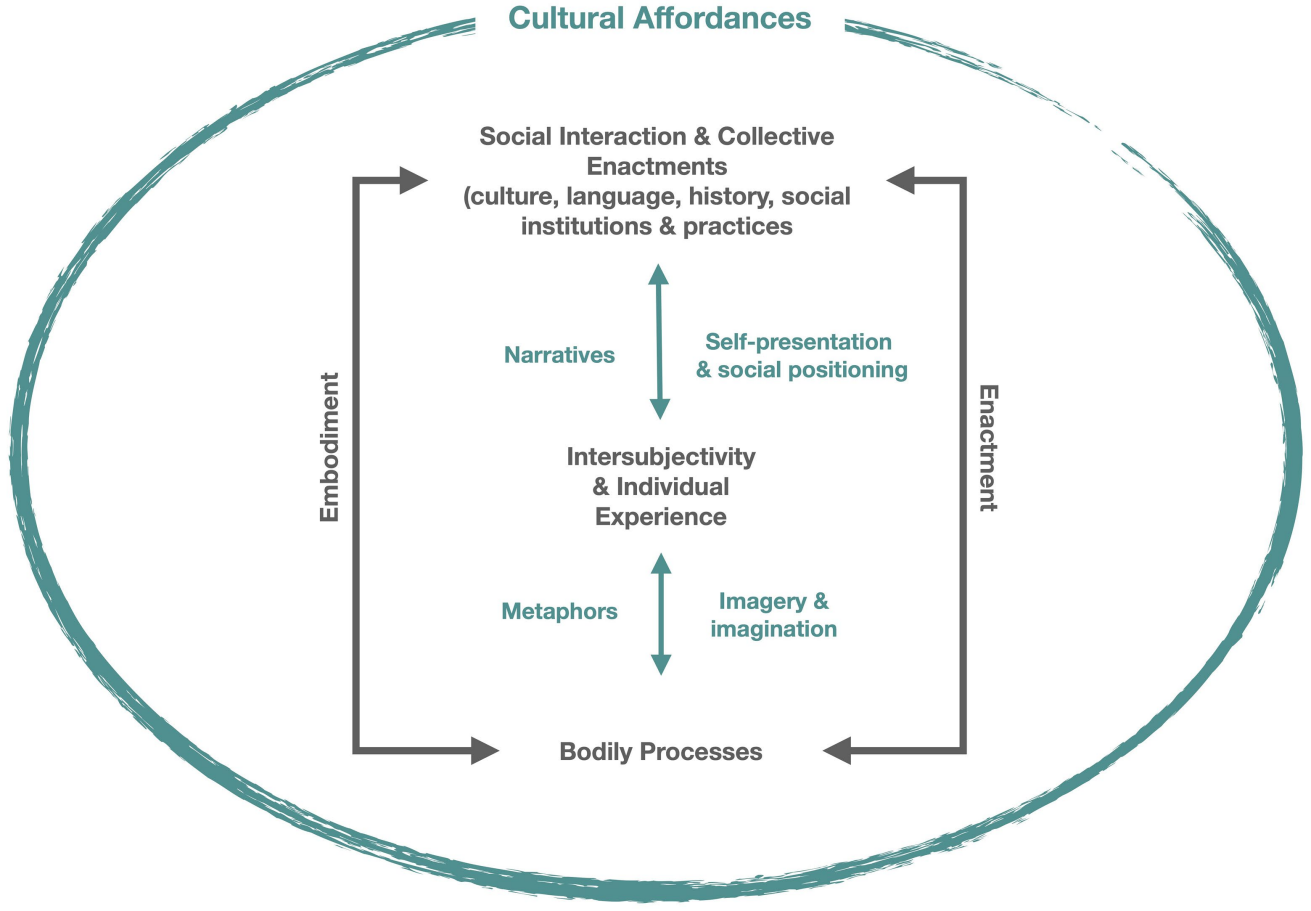
Embodied and Enactive Processes of Meaning Making. The figure outlines the cyclical processes of embodiment and enactment that give rise to meaning and experience. Experience emerges through developmental processes and engagement with others in particular social-cultural contexts. There is two-way traffic between bodily processes and individual experience mediated by cognitive processes of metaphoric thinking, imagery and imagination. Similarly, there is traffic between experience and social discourse mediated by interpersonal communication and narrative practices. All of this occurs in a field of cultural affordances provided by local niches and larger social contexts (Adapted from Figure 20.1 in (204); originally published in *Embodiment*, *Enaction*, and *Culture*: *Investigating the Constitution of the Shared World*, edited by Christoph Durt, Thomas Fuchs, and Christian Tewes, reprinted courtesy of The MIT Press).

Many of the links shown in Figure 1 are mediated by personal, social and cultural processes of meaning making. These involve bodily and discursive practices as depicted in Figure 2. While physical stressors may have direct effects on physiology and elicit responses, based on past experience, that occur outside of awareness, the impact of stressors also depends on individuals’ perception and interpretation of the event. This involves embodied and enactive processes of meaning-making that build on developmental experiences and draw from cultural resources (204). The process of meaning-making includes the person’s appraisal of the level of threat, their coping skills and resources, and the potential consequences—that is, “what’s at stake” for the individual and others in their social world (238). For example, while job loss is likely to be a stressor for most people, the degree of perceived stress and ability to cope will depend on contextual factors including the personal and cultural meanings of one’s occupation and of unemployment, current economic resources, social supports and mobility.

Shame and humiliation follow from experiences of loss of social status and failure in performing according to social norms (239). The experience of humiliation in response to job loss depends on its timing (e.g., family just moved for the job or has had other resource depleting stressors), social position, roles, norms and expectations (e.g., father expects to be a breadwinner). Social validation of perceived stress can also contribute to self-regulation and reduction of perceived stress through process of feeling understood, supported and protected, as well as helping the individual to shift perspectives, mobilize problem solving strategies, and access stress-reducing resources.

Perceived stress can prompt multiple maladaptive behaviors that feedback in loops that lead to resource depletion. For example, drug consumption for symptom control, relaxation and or escape can lead to emotional lability and irritability that challenge relationships. In favorable constellations however, response to perceived stress may lead one to develop new skills or positive schemas, overcome engrained biases, rescript self-understanding narratives, expand one’s affordances, deepen social relationships and improve coping.

In the ecosocial systems view, interpersonal dynamics, work stress, gender discrimination, and cultural knowledge and practices for dealing with distress—all of which depend on or reside primarily in social interactions—may contribute to the patient’s distress, coping strategies and process of recovery. Applying an integrative perspective in case formulation requires considering how these processes unfold over time in the individual’s life trajectory. Moreover, the processes related to each of these levels and dimensions interact in ways that can give rise to feedback loops that exacerbate symptoms and result in a depressive disorder or other syndrome, which may then be maintained through similar looping mechanisms (237). These loops are not only internal to the brain and its circuits but extend beyond the body to social interactions with other people and social institutions—all of which affect the development and course of psychiatric disorders.

The cultural-ecosocial approach is fundamentally relational. The relationships it considers involve material, informational and symbolic-communicational interactions between the individual and the environment. These relationships can be mapped by causal loop diagrams (CLD) that aim to capture the links between observable processes (240). These maps can be used to develop formal quantitative models to reveal dynamics and test the potential impact of interventions, including changes in the configuration of systems—e.g., by altering individual biology or cognition, family interactions, health care systems or other social contingencies (241, 242).

In the ecosocial view, humans are embedded in and dependent on culturally constructed environments that include physical arrangements as well as a web of relationships with other people and social institutions. The 4E perspective insists that interactions with the environment are part of the dynamics that constitute the individual. In human ecology, however, the distinctions between individual and environment are phenomenologically, psychologically, morally and politically important. Hence, drawing the boundary between ‘inside’ and ‘outside’ (organism and environment or system and subsystem) varies with the clinical question and the way we locate the relevant dynamics (243). There can be principled and practical reasons for drawing a boundary in a particular way both because it highlights crucial dynamics and constitutes a useful way to organize case formulation and guide intervention. These reasons may include the system’s topology, the feasibility of specific interventions, and the ethical imperative to privilege the patient’s perspective (244).

## Integrating the patient’s self-understanding

A key element in an ecosocial systemic approach is recognizing the role that the person’s own understanding of and response to symptoms and suffering play in the dynamics of mental disorders, coping, help-seeking, treatment response and recovery. In the case vignette presented in the previous section,the patient’s self-diagnosis and explanatory model of her symptoms follow closely from the prevailing brain-centric model of depression widely disseminated in popular culture. This model portrays depression as a condition related to specific neurotransmitters and explains the efficacy of medications by their effects on corresponding receptor sites. More recent versions of this explanatory model go beyond synaptic mechanisms to consider brain circuitry (245–247). Other patients may present explanations that draw from sociomoral or religious understandings of suffering and view illness as a consequence of moral transgression or failing (248). These modes of explanation and attributions influence ways of coping and help-seeking but they may also participate in the vicious circles that aggravate dysphoria, self-deprecation, social withdrawal, and other symptoms of depression (249).

The patient’s illness narrative, which emerges in dialogue with available cultural models and in clinical encounters, also shapes the process of meaning-making and illness experience (238). The models used by clinicians — which borrow from both technical literature and dominant cultural narratives — also shape patients’ experience and expectations (173). In this case vignette, the patient adopted a simple biological model of depression even before speaking to the doctor, setting aside her challenges of job loss and relationship problems as secondary issues. In so doing, she focused her expectations in consulting the clinician on receiving a specific medication. While this fits squarely with psychiatrists’ competence, it may require negotiation, because her self-diagnosis may not be accurate and her requested treatment may not be appropriate, and, even if it does address an important facet of her current problem, medication may not be sufficient to resolve other aspects of her predicament (250, 251).

The effects of adopting a neurobiological explanation go beyond a narrow focus for clinical assessment and treatment to also influence the patient’s sense of self-efficacy and participation in the process of recovery as well as broader features of her identity. A simplified, brain-centric model of depression makes antidepressant prescription seem a straightforward, necessary, and sufficient clinical response. Of course, beyond pharmacogenetics, kinetics, and dynamics, our mechanistic knowledge of drug action remains limited (252). Antidepressant treatment may have different effectiveness based on the individuals’ expectation of efficacy (253) or their socioeconomic status (254), requiring the clinician to consider the interaction of the type of treatment and the patient’s context when collaboratively designing a care plan (255). Moreover, prescription is inevitably a social and symbolic act, and taking medication has meaning and consequences for psychological self-regulation and social identity (256, 257). Rose (258) has drawn attention to the ways that biomedical diagnosis and treatment of mental disorders lead to narratives of “neurochemical selves” with consequences for individual coping as well as for mental health policy and practice. There is increasing recognition that good practice in psychopharmacology requires paying attention to the personal and cultural meanings of medication and patients’ own values and priorities (259). A cultural-ecosocial view can inform existing approaches to shared decision making and collaborative prescribing or deprescribing of medication (255).

## An ecosocial systems approach to person-centered clinical practice

Psychiatric practice employs multiple ways of knowing that have been characterized as *verstehen* (understanding), *erklären* (explaining) and *einfühlung* (empathic, embodied co-presence/being/knowing) (260). These ways of knowing have different epistemic bases and constraints and are sometimes in tension, conflict or competition. In contemporary psychiatry, this tension is seen between the divergent approaches of precision psychiatry (which characterizes the person in terms of biological parameters) and person-centered psychiatry (which emphasizes experience, values and context) (261, 262). Although advocates of each approach superficially acknowledge the other, in practice their respective research programs and modes of implementation reflect the persistence of an underlying dualistic ontology (129, 218). Bringing *erklären*, *verstehen*, and *einfühlung* together in clinical formulation means integrating explanatory models and mechanisms across levels, including molecular, physiological, neural circuitry, cognitive, and social. Including the social level requires knowledge of social and cultural history and current context as well as biographical trajectories. Because our institutions and practices are embedded in these same contexts, a social-cultural perspective requires self-reflective consideration of the clinician’s positionality and interaction with the patient and others in the co-construction of clinical narratives (260). The cultural-ecosocial systems approach offers a frame that can encompass these dimensions of psychiatric practice through a dialogical process of meaning-making that recognizes culture and context.

Human ecological niches are fundamentally *social*—with socially constructed contexts and relationship providing the essential matrix of development from inception—and *cultural*, with shared meanings, values and practices shaping cognition and experience across the lifespan. The notion of ecosystem builds on work in ecological systems theory in developmental psychology (68), which emphasizes the embedding of the individual in multiple, nested environmental contexts, defined by socio-relational and spatio-temporal scale and composition to include: *micro* (immediate family and friends, community and work-school setting); *meso* or *exo* (neighborhoods, wider networks, and larger community); and *macro* (society, nation, transnational) contexts. (See: Table 1), The idea of a niche highlights the interactive and dynamic nature of such sociocultural embedding. Social context, structural, economic and political forces affect individuals and groups differentially as a result of individual and collective past histories, biology, and current positionality (263).

To unpack the notion of niche in a way that can serve a person-centered clinical approach, the ecosocial systems view needs to consider the intersections and interactions across at least four over-lapping domains: (1) lifespan developmental history; (2) social structure and positioning; (3) cultural meaning, norms, values and affordances; and (4) individual biography and self-understanding (which draws selectively from each of the other domains). These domains can provide a temporal dimension to clinical formulation that points both to adaptive challenges and resources for helping, healing and recovery. Efforts to develop models that incorporate social context and lived experience are underway, but they face multiple obstacles, including lack of collection of data representative of population variability and high levels of context dependence as well as ethical and pragmatic issues related to the use of such data (264). We need better conceptual, research and clinical tools to characterize niches—their demands, affordances, and constraints as well as their embedding in larger ecosystems (6). The theory of syndemics provides one approach to exploring the multilevel interactions that give rise to mental health problems (265, 266).

While the notion of niche points to the immediate environment that an individual inhabits, in reality, human niches are subsystems of larger social systems. An ecological view encourages us to examine this larger network of relationships and how they interface with local niches. It is a virtue of the ecological perspective that it allows us to think systematically about the relationships between our most proximal and intimate relational networks and the larger networks with which we are coupled. The nature of this coupling depends on local arrangements and interpersonal interactions, which are extended by population migration as well as information and communication technologies that allow connections with distant others but that also create virtual environments that we increasingly inhabit (267, 268).

In the current moment, relationships on the planetary scale are increasingly present and consequential in the lives of individuals through the impacts of climate change (269, 270). These interactions occur in material ways, but they are also present in self concepts, imagination and orientation toward the future with significant mental health impacts. True to its name, an ecosocial view, encourages us to think about mental health as dependent on these wider networks and modes of interdependence. Coming to terms with the impact of our changing environments requires considering not only strategies for individual adaptation, but the larger, social structural arrangements that account for global disparities and that constrain the options of individuals and groups across the globe (271, 272). Ultimately, mental health theory and practice must consider not only the private challenges of individuals, but the larger dilemmas faced by our species and the planet we share with others (273).

## Conclusion

Although psychiatry conventionally locates mental health problems in the individual, systems thinking encourages to see the ways in which health and the wide range of problems seen in clinical settings arise from interactions at multiple levels from the biological to the cognitive and social. Recognizing patients’ agency and restoring their health requires that clinical care consider the range of systemic processes that contribute to suffering and impairment (274). Addressing problems that derive from social structure may require interventions that go beyond individual clinical care to include advocacy and social-network interventions. Advocacy is not limited to efforts to change policy and institutional practices but includes actions that aim to counter oppressive circumstances and create habitable environments and niches for individuals (275).

Efforts to provide multilevel systems explanations of health problems are often challenged as “too complex” for practical application. Systems dynamics may be difficult to think through and require specific training to apply. Complex systems can exhibit counterintuitive properties, but qualitative understanding is often sufficient to guide practice (276–279). Quantitative models of specific problems could allow clinicians to examine the effects of potential interventions on system dynamics to guide treatment and predict outcomes. Crucially, these models can include clinician-patient interaction and other social processes as part of the symptom network. Innovative computational methods can capture multilevel system dynamics if the relevant data are collected (264). The resultant models could be used as decision tools or used by clinicians and patients to foster mutual understanding and motivate interventions. The models we offer to patients are themselves interventions that may guide self-reflection and elicit new behaviors. They may also function as self-fulfilling explanations that foreclose the search for better answers. How this plays out depends on the ability of the clinician to apply dynamical systems models while closely attending to the patient’s experience so that the model can be refined and care remains patient-centered.

The application of dynamical systems models in psychiatry, though actively pursued for decades, has been slow to advance and has had limited uptake. There are several likely reasons for this, including that the adoption of systems thinking has been hampered by (i) continued investment in reductionist models because they are amenable to study by common scientific methodologies; (ii) the limitations of clinical decision making, which make it hard to incorporate complexity and interaction effects; and (iii) economic and political interests that favor short-term treatment and pharmacological interventions rather than approaches that challenge entrenched systems. However, new computational modelling methods that can be implemented in clinical settings to support patient education and real-time decision making offer the hope of significant progress.

The challenges associated with complexity reflect the real-world dynamics of human problems (280, 281). Recognizing this complexity should urge on us humility and the need to frequently recalibrate our clinical response to respond to patients’ experience. It underscores the need for idiographic methods of case formulation, which may include characterizing networks of relationships among symptoms and related biological, cognitive, and social processes (194, 282). Finally, it points to the importance of self-reflexivity, in which clinicians interrogate their own assumptions and practices to rethink case formulations and potential interventions.

The cultural-ecosocial view includes practitioners, clinical settings, health care systems and the local and international institutions of psychiatry itself — both as material and discursive practices— as part of the systems in which patients and practitioners are embedded and which offer them affordances, norms and constraints. These need to be factored into practice in general and into the formulation of specific cases. A literature in critical psychiatry has considered some of the ways in which psychiatry colludes with larger structures of oppression (201, 283). This is more likely to occur when psychiatric practice is narrowly conceived as the identification and treatment of discrete disorders without attention to patients’ lived experience, values, and lifeworlds as well as to practitioners’ tacit assumptions. By giving an explicit place to the meaning-making process in clinical encounters as well as in institutional and wider social contexts, a cultural-ecological systems view opens the door to more self-reflective and critical thinking that can uncover power dynamics and counter potentially oppressive practices.

An ecosocial systems view offers a way for clinicians to organize the multiple explanatory models needed to capture the complexity and heterogeneity of psychiatric disorders and illness experience. Based on a view of psychiatric disorders as involving complex system dynamics, an ecosocial systems approach allows clinicians to use multiple languages of description to assess processes within and across levels of organization of an overarching ecology of mind and to prioritize those that offer the greatest therapeutic leverage and optimal use of resources for person-centered practice.

## Data availability statement

The original contributions presented in the study are included in the article/supplementary material, further inquiries can be directed to the corresponding author.

## Author contributions

AG-C and LK contributed equally to conceptualizing the manuscript. AG-C wrote the first draft. LK wrote sections of the manuscript. All authors contributed to the article and approved the submitted version.

## Funding

Work on this paper by AG-C was supported by a Banting Fellowship from the Canadian Institutes of Health Research. LK received project support from the McGill-CFREF Healthy Brains for Healthy Lives Program through a grant for the development of a Canadian Framework for Brain Health (3c-KM-61).

## Conflict of interest

The authors declare that the research was conducted in the absence of any commercial or financial relationships that could be construed as a potential conflict of interest.

## Publisher’s note

All claims expressed in this article are solely those of the authors and do not necessarily represent those of their affiliated organizations, or those of the publisher, the editors and the reviewers. Any product that may be evaluated in this article, or claim that may be made by its manufacturer, is not guaranteed or endorsed by the publisher.

## Footnotes

^1^There is no doubt that notions of hierarchy reflect sociomoral and political values (73), but this does not vitiate their use as technical concepts in science or other domains.

^2^Of course, there is legitimate concern about the need to recognize oppressive structures in society but these are not simply due to hierarchically structured systems. Oppression can arise not only from the dominance of one group over others but from systemic processes that occur at multiple levels in the system. For example, collective norms and public discourse can legitimate discrimination, micro-aggressions and social exclusion with significant effects on the health of minorities. Hence, it is important to distinguish between hierarchical structure as an organizational feature of complex, self-organizing, goal-oriented systems and oppressive processes that make use of hierarchies, but also other aspects of social structure and everyday practice, to exert power in ways that create inequity. Ecosystems and social systems might be better characterized in terms of the concept of "panarchy" in the sense that they exhibit both top-down and bottom-up causation often on different spatial and temporal scales (78). The potential for conflict between these levels of causation and control leads naturally to a consideration of the dynamics of social power.

^3^We use the term “teleodynamic” in a way similar to Deacon (99), who contrasts *teleonomic* systems that can be *interpreted* as pursuing a goal state, from *teleodynamic* systems that actually pursue goal states as basic to their structure (see 100, 101).

^4^Multicellular organisms regulate and “police” their own constituents in ways that contribute to the survival of the whole organism (through allostasis and reproduction) at the expense of the viability of individual cells (106).

^5^We use the term ‘translate’ here deliberately, not only to capture the fact that higher order neurocognitive processes involved in language mediate the effects of social stimuli on physiology (e.g. 133) but also, because in responding to symbols and situations the brain must "translate the relations between single elements of a given situation (stimuli) into wholes". The dynamics of cognitive systems involve the regulation of information, which resides in the relationship of organism to context (﻿^134^﻿). Human systems have both dynamic and linguistic modes that require corresponding descriptions. Our self-descriptions, narratives and metaphors, on this view, participate in the dynamics of adaptive systems but to do so, they require a translation (i.e. a meaning- and context-sensitive mapping) from the pragmatic communicative situations of linguistic communication (and representation) to the dynamics of brain systems and physiology.

^6^For definitions of these terms and others used to characterize system dynamics, see: Mainzer (21); for examples of how they may be related to specific types of pathology; see: Durstewitz et al. (132).

^7^The term *embodiment* is also used in Krieger’s (188) ecosocial theory in population health to stand for the ways in which the social environment "gets under the skin" to affect physiology. These processes are central to understanding the social determinants of health. However, the notions of embodiment in 4E cognitive science draw from phenomenology (67) and cognitive-social psychology (150) to give an account of the process of sense-and meaning-making that can clarify the nature of illness experience and coping (1, 186, 189).

^8^This case vignette is a fictional composite based on the authors’ clinical experience designed to illustrate cultural-ecosystemic formulation.
